# Supplementary material for: Learning curve and surgical outcome of robotic assisted colorectal surgery with ERAS program
Source: Sci Rep. 2022 Nov 29;12:20566. doi: 10.1038/s41598-022-24665-w (PMC9709162; doi:10.1038/s41598-022-24665-w)
Supplement: Supplementary file 1 — Supplementary Information 1. [file 41598_2022_24665_MOESM1_ESM.zip › LSC Raw data-Ñ[▒K/095/h_ñw╝╨░O▒Kñσ.pdf]

正式摘要(電子簽章：000000206187179)

W55 / W55 ☐ 英文標題(注意:無英文姓名-請補病人基本檔!) ☐【讀取】憑證

臺中榮民總醫院

出院病歷摘要

轉入號： 轉出號：

[redacted]

(8)流水編號	
(9)入院日期	109/09/15 (2020) 直腸外科 W55 - 261
(10)轉科日期	
(11)出院日期	109/12/22 (2020) 住院天數計 98 日

診斷

- 入院：1. Right side T colon cancer, cT3N1M0
  2. GBS
  3. T2DM
  4. CAD-III post successful PCI of distal RCA to PDA proximal (POBA+DESX1) , of LAD proximal to middle BMS ISR (POBA+cutting+DEBx1) on 2020/01/06
  5. Congestive heart failure(2020/09/18 ECHO: LVEF 41%)
- 出院：1. Right side T colon cancer, cT3N1M0, s/p laparscopic right hemicolectomy + D3 dissection on 2020/09/30, c/w anastomosis leakage, s/p exploratory laparotomy + ileostomy on 2020/10/14.
  pT3N0(0/32)M0, pMMR, MD, AI-, PI-, TdDp-, MacPf-, CRM:1.5cm, stage IIA
  2. GBS s/p LC on 20201004
  3. T2DM
  4. CAD-III post successful PCI of distal RCA to PDA proximal (POBA+DESX1) , of LAD proximal to middle BMS ISR (POBA+cutting+DEBx1) on 2020/01/06
  5. Congestive heart failure(2020/09/18 ECHO: LVEF 41%)

主訴

- dizziness and vertigo since 09/13 08:00

病史

- The 71 y/o man, with past hx of
  (1) DM(hypoglycemia happened before);
  (2) CAD-III post successful PCI of distal RCA to PDA proximal (POBA+DESX1) , of LAD proximal to middle BMS ISR (POBA+cutting+DEBx1) on 2020/01/06
  (3)Congestive heart failure(108/12/10 ECHO: EF 28%);
  (4) OA;

  This time, he suffered from dizziness and vertigo since 09/13 08:00, bloody and tarry stool was also noted recently. He denied fever, chills, headache, dizziness, URI, dyspnea, chest pain, diaphoresis, N/V, abdominal pain, diarrhea, dysuria, or focal neurologic deficits was noted this time.
  At our ER, Hb showed 8.3, no PT/aPPT prolong. Upper GI scopy showed reflux esophagitis, LA, Gr A; Antral shallow ulcers; no stigmata of recent bleeding. L-SCOPY showed one tumor at 60 cm from AV suspect maligancy s/p biopsy and hemoclip marking. Abdominal CT showed R/I colon cancer at hepatic flexure with pericolic invasion and regional lymphadenopathy. Under the impression of R/I colon cancer, he was admitted for further management.

體檢發現

- RV (3.1 CM) & LV (5.8, 5.0 CM) CHAMBER DILATATION
  CONC. LVH (1.2, 1.2 CM)
  AORTIC ROOT DILATATION (3.9 CM)
  AORTIC VALVE THICKENED WITH MILD AR
  MITRAL ANNULAR CALCIFICATION WITH MILD MR
  MILD TR WITH PEAK/MEAN SYSTOLIC PG – 32/23 MMHG
  DILATED PA TRUNK (– 2.6 CM) WITH MINIMAL PR
  REVERSE MV E/A RATIO
  THE LV EJECTION FRACTION IS 28 %
  THERE IS GENERALIZED LV HYPOKINESIS, ESP.
  THE LV OF DISTAL 1/2 IVS TO APEX & DISTAL 1/2 INFERIOR WALL & INFEOLATERAL WALL WALL SHOWED AKINETIC MOTION
  MV E/E' SEPTAL– 8.61
  MV E/E' LATERAL – 5.31
  END
  - General appearance :
    - easy looking , conscious clear , without cardiopulmonary distress
    - Vital signs門診帶入date 體溫(BT) 脈博(P) 呼吸(R) 血壓(BP) 身高(BH) 體重(BW) BMI
      20200915 35.5℃ 92次/分 17次/分 118/59mmHg 161.1cm 68.4kg 26.4
    - Vital signs: B.P. 118/59 mmHg , P.R. 92 /min , R.R. 17 /min , B.T. 35.5 ℃
  - HEENT :
    - Eyes: no anemic conjunctiva, no icteric sclera, normal EOM
    - ENT: no otorrhea, no rhinorrhea, no erythematous mucosa, no injected tonsil, no deformity
  - Neck: supple, freely movable, trachea midline,no deformity, no engorged jugular vein , no palpable thyroid or lymph node enlargement
  - Chest:
    - Inspection: Grossly normal, symmetric and fully expansion, no accessory muscle used, no operation scar, no spider angiomata, no gynecomastia
    - Palpation: no palpable mass or nodule, no subcutaneous emphysema, no tenderness
    - Ausculation: clear breathing sound
  - Heart:
    - Palpation: no thrill, no heave
    - Percussion: normal heart size
    - Ausculation: regular heart beat, normal S1, S2, no gallop, no murmur
  - Extremities: No deformity, full ranged of motion without limitation, no clubbing finger or nail cyanosis, no pitting edema, no ulcer or pigmentation, no obvious skin lesion, no varicose vein, no bone, joint or muscle tenderness

  CRS CONDITION
  - Karnofsky performance status: (100) Normal, no complaints, no evidence of disease
  - PE of Abdomen:
  - __1. Inspection: normal
  - __2. Ausculation: hyperactive
  - __3. Palapation: no tendenrness
  - __4. Percussion: dullness
  - CEA: 0.959 ng/ml
  - Imaging Studies:
  - Colonoscope report: One tumor at 60 cm from AV suspect makligancy s/p biopsy and hemoclip marking
  - Figures:
    1. colon & rectum

手術日期及方法

- 手術日期及方法20201014 Exploratory laparotomy
  20200930 Laparoscopic right colectomy+ D3 dissection
  20200930 Laparoscopic Cholecystectomy

住院治療經過

- After admission, Transamin and pantoprazole was prescribed. Tarry/Bloody stool subsided. Abdominal CT r/i colon cancer at hepatic flexure with pericolic invasion and regional lymphadenopathy. Lung CT showed no evidence of lung metastasis. Liver MRI showed hepatic flexure colon cancer and small hepatic cyst. FVC: FEV1/FVC 79%. 2D echo showed LVEF 41%.

- We also consulted CV doctor for DAPT medication adjustment, suggested hold 5 days before operation. DAPT was hold since 9/26, and add back on 10/5. The patient underwent operation on 2020/09/30. The operation was done smoothly. After the surgery, TPN and 0.45% G/S 1000 c.c. QD was given for fluid supplement and nutrition support. However, dyspnea was noted on 2020/10/04. His body weigh increased (68.4 -> 73.3 kg). Bilateral leg edema 1+. Artery gas showed pH: 7.405, pO2: 79.7, pCO2: 37.9, HCO3: 23.2. CXR showed bilateral lung infiltration. CHF related fluid overload was suspected, s/p Rosis 40mg Q8H mixed with Albuminar injection. IV fluid was titrated. His dyspnea gradually improved. Also, the patient complained about abdominal distention. X-ray showed distended stomach, s/p NG tube decompression during 10/5-10/12. He started to try Bland I diet since 10/12. On 10/11, dark red discharge was noted from the lower abdominal wound. We removed the stitch with BID wet dressing of the wound. The pus culture collected on 10/11 yielded Escherichia coli. We re-checked abdominal CT on 10/14. Loculated fluid accumulation with wall enhancement at right subphrenic space, right subhepatic space, right paracolic gutter, bilateral mesentery and pelvic cavity is mentioned, in favor of abscess formation. Thus, exploratory laparotomy was performed on 10/14 for anastomosis leakage. After the surgery, he was transferred to SICU for further care. In SICU we kept the inotropes first for septic shock status. Micafungine was perscrobed for suspect fungal infection. We changed his CVC on 10/15, and obtain blood for culture. Abx regimen was shift to Tazocin after discussed with ID specialist. We let him tried water on 10/17, and the digestion was good, so diet was giiven on the same day. CXR found LLL or LML atelectasis, so we consulted CS for bronchoscope on 10/20, and found sputum impaction in the bronchus. Sputum was removed, and sent for S/C. Tarry stool was noted on 11/1. Hb also dropped to 7.8g/dL, so PRBC 2U was transfused. UGI scope and abdominal CT was arranged on 11/2 that the result showed duodenal ulcer without active bleeding. Thus, we kept PPI and PPN support. Tarry stool was not noted after. Ascites culture yielded Kp and E. coli growth, so antibiotics was shifted to Cefepime on 11/4. We also let him try water and then increased feeding amount. Endotracheal tube was removed on 11/8. Under the relatively stable condition, he was transferred to ordinary ward on 11/11. At our ward, we started removing Foley-sump drain 2cm each day from 11/14 and finally removed it on 11/22. His appetite gradually improved and we We consulted Rehab on 11/23 due to bil. leg weakness. We also consulted DERM due to his itchness complain with rash over face, upper back, chest and arms. They implied the suspicion of mililaria rubra ir allergic dermatitis. We use Mycomb and Xyzal as they suggested. He felt partial improved. We tried Foley's catheter removal on 11/18, 11/24 but failed. We will keep current treatment and preparation for MBD.

合併症

一般檢查

- 累積報告：

DATE	TIME	ALB	TP	BIL.T	BIL.D	ALKP	AST	ALT	LDH	CHOL	TG	HDL-C	LDL	UA	NH3	AMY
1090913	1100							6								
1090913	1753			0.3					109							
1090917	0938	2.8					18	7		114	74					
1090921	0925	3.0					11	9								
1090924	0915	2.9					12	7								
1090928	1100	2.8					12	9								
1090930	1943	2.6		0.37	0.13	68	29	17								
1091004	1437					80	33	4								
1091005	0654	2.2														
1091008	0929	2.8		0.50	0.25		13	3								
1091010	1447															22
1091012	0935	2.9		0.46			16	3								
1091014	1157	2.7		0.25	0.14		30	3								
1091015	0049	1.8		0.21				3								
1091015	0230			0.3	0.1											
1091015	0726			0.29	0.15		18	4	199							
1091017	0547			0.73	0.31		23	4								
1091018	0533	2.6														
1091021	0526			0.6	0.3	128	14	5								
1091024	0518	3.3		0.66				14								
1091026	0529			0.52	0.21	142	34	22								
1091027	0515			0.5	0.2		24	19								
1091028	0510			0.4				22								
1091030	0608			0.39												
1091101	0511			0.34	0.15		24	28								
1091103	0454			0.62	0.25	169		28								
1091105	0600			0.39	0.19		32	32								
1091108	0508	3.6		0.26	0.14	189	28	31								
1091110	0521			0.2	0.1	216		35								
1091114	1748			0.2	0.1	180	26	35								
1091119	0932	3.6		0.2	0.1		19	23								
1091124	0922	3.5		0.19	0.11		25	8								
1091127	0852	3.5		0.17	0.09		24	10								
1091130	1310	3.6		0.39	0.17		39	28								
1091203	0909	4.0		0.4				39								
1091210	0930	3.5		0.4				44								
1091218	0928	3.5		0.3				32								

DATE	TIME	WBC	RBC	HGB	HCT	MCV	PLT	BLST	NEUT	BAND	SEG	LYM	MON	EOS	BAS
1090913	1100	11080		8.3			414		74.9			14.5	7.1	3.2	0.3
1090913	1753			8.2											
1090914	0510			10.0											
1090915	0539			10.9											
1090917	0912	7160	3.79	9.5	31.8	83.9	366		71.6			16.3	9.1	2.7	0.3
1090921	0901	8940	3.95	10.5	32.3	81.8	289		71.0			15.9	8.8	4.1	0.2
1090924	0857	11900	3.76	10.0	31.2	83.0	312		72.2			14.7	8.3	4.5	0.3
1090928	1054	12960	3.75	10.3	31.5	84.0	345		77.0			12.6	6.9	3.3	0.2
1090930	1943	17020	3.81	10.3	32.1	84.3	308		91.8			4.4	3.5	0.1	0.2
1091004	1433	28360		10.6		85.4	343			1.0	93.0	2.0	4.0	0.0	0.0
1091008	0912	35040	3.54	9.9	30.1	85.0	381			3.0	88.0	3.0	6.0	0.0	0.0
1091012	0912	23020		10.6		85.9	470				83.0	3.0	8.0	2.0	0.0
1091014	1155	18640	3.56	10.0	29.9	84.0	470				83.0	6.0	10.0	1.0	0.0
1091015	0051	29000		9.7	29.9		522			1.0	92.0	3.0	2.0	1.0	0.0
1091015	0228	36670	3.79	10.2	31.7	83.6	550		94.7			2.3	2.6	0.2	0.2
1091015	0714	43300	3.73	10.2	31.9	85.5	661				93.0	2.0	4.0	0.0	0.0
1091016	0522	30490		7.7	23.6		528				87.0	5.0	8.0	0.0	0.0
1091017	0547	23410	3.05	8.9	26.4	86.6	421		87.1			6.2	4.7	1.8	0.2
1091018	0533			8.9	27.9		392								
1091019	0557	13650		8.7	27.0		415		85.3			6.9	5.6	2.1	0.1
1091020	0552			9.0	27.6										
1091021	0524	17620	3.13	8.9	27.7	88.5	445		90.7			6.0	3.2	0.0	0.1
1091022	0531	13340		9.9	31.2		415		85.5			8.7	5.5	0.2	0.1
1091024	0513	14830	3.56	10.2	31.5	88.5	428		89.8			6.9	3.2	0.0	0.1
1091026	0528	13480		10.1	30.7		438		82.8			9.3	6.4	1.3	0.2
1091027	0515	14110	3.42	9.7	30.3	88.6	438		86.1			8.5	4.3	0.9	0.2
1091028	0510	13000	3.37	9.5			413		81.5			8.8	8.0	1.5	0.2
1091030	0606	14460		9.3	28.5		410		81.6			9.1	7.8	1.3	0.2
1091101	0512	19310	2.71	7.8	24.4	90.0	393		86.6			6.7	6.2	0.3	0.2
1091101	1821			8.8	26.8										
1091102	0509			7.6	23.6										
1091102	1558			9.8	28.3										
1091102	2311			9.1	27.6										
1091103	0454	12880		9.2	26.8		326		80.9			10.9	7.0	1.0	0.2
1091103	1511			9.8	27.5										
1091104	0514			9.2	27.0										
1091105	0555	11830		9.3	27.7		368		81.7			9.7	6.5	1.9	0.2
1091108	0508	9400		8.8	26.6		368		75.0			14.5	7.1	3.1	0.3
1091110	0520	10900		9.2	28.9		415		84.1			9.8	5.9	0.1	0.1
1091114	1748	9050	3.56	10.8	33.2	93.3	370		76.9			13.0	5.6	4.3	0.2
1091119	0922	10990	3.46	11.0	33.0	95.4	279		79.0			12.6	5.5	2.6	0.3
1091124	0903	7210	3.76	11.8	34.8	92.6	235		68.1			22.3	7.4	1.9	0.3
1091127	0857	6360	3.56	11.4	33.2	93.3	247		65.4			21.9	7.2	5.3	0.2
1091130	1319	7410	4.05	12.9	38.4	94.8	270		73.7			17.5	5.0	3.5	0.3
1091203	0923	7380	4.15	13.1			320		73.0			20.2	4.9	1.6	0.3
1091210	0933	6070	3.68	11.6			261		69.3			23.4	5.6	1.5	0.2
1091218	0906	7180	3.79	12.2			249		74.1			19.1	5.0	1.5	0.3

DATE	TIME	NA	K	CL	CA	BUN	CREAT	Mg	P	CRP	ESR	PCT	LIPASE	LACTATE
1090913	1100	137	5.1			30	1.02							

-

1090917	0938	138	4.4	106	7.3	8	0.90	2.2	2.8					
1090921	0925	134				24	0.88							
1090924	0915	135	4.5		7.9	25	0.96	2.1	3.8					
1090928	1100	135	4.5		7.9	29	0.98							
1090930	1943	137	4.6	102	7.1	23	0.87							
1091004	1436													12.1
1091004	1437	125	4.5			24	0.75							
1091008	0929	131	3.0		7.0	27	0.99	1.8						
1091012	0935	134	3.4	90	7.4		1.03							
1091014	1157	131			7.1	27	1.00							
1091015	0049	131	3.3	94	6.6	24	0.91							
1091015	0230	131	3.4				0.99							
1091015	0723													39.2
1091015	0726	131	4.2	92	7.0	29	1.22							
1091016	0511	132	3.8	95	7.1	34	1.66							
1091016	0513													15.8
1091017	0544													8.1
1091017	0547	131	3.9	96	7.0	30	1.79							
1091018	0533	129	4.1	96	7.1		1.64							
1091019	0600	132	4.2											
1091020	0556	134	4.3			20	1.57							12.3
1091021	0524													10.9
1091021	0526	136	4.6	102	7.4	22	1.65							
1091021	1854					25	1.72							
1091022	0530	135	4.0	103	7.6									
1091024	0514													8.7
1091024	0518	140	3.3		7.5	28	1.67	1.8	2.7			0.205		
1091025	0553		3.6											
1091026	0529	139	3.8	107	8.1	23	1.52							
1091027	0515	142	4.2	107	8.7	24	1.43							
1091028	0510	142	4.3		8.5	30	1.45							
1091030	0608	137	3.8	102	8.4	43	1.38							
1091101	0509													17.8
1091101	0511	136			8.3	58	1.38							
1091102	0509		4.4											
1091103	0454	131	4.2	101	8.6	51	1.31							
1091104	0511											0.107		
1091104	0515													8.5
1091105	0600	131	4.5	100	8.9	36	1.29							
1091107	0510	128	4.6											
1091108	0508	130	4.7	102	8.8	45	1.15							
1091110	0521	136	5.5	108	8.8	35	1.05							
1091110	1505		5.1											
1091111	0509		4.8											
1091114	1748	139	4.7		9.0	33	1.01			0.176				10.8
1091119	0932	140	4.2		8.5	23	1.01							
1091124	0922	140	4.0		7.9	23	0.90							
1091127	0852	137	4.0		8.0	21	0.96							
1091130	1310	140	4.1		8.7	24	0.99							
1091203	0909	140	4.5	107	9.0		1.13							
1091210	0930	141	4.5	109	8.6	33	1.29							
1091218	0928	140	4.1	107	8.3	25	1.15							

DATE	TIME	GLUCOSE	PAB	TRF	ZN	CU	KETONE	CK	CKMB	PT-P	PT-C	APTT-P	APTT-C	TRO-T
1090913	1059											28.7		
1090913	1100	76												
1090913	1752									11.2	10.3			
1090913	1753	92												
1090928	1053									11.1	10.3	35.7		
1090930	1943	189												
1091004	1436													11.47
1091004	1437							20	3					
1091024	0514													33.01
1091024	0517								3					
1091024	0518							11						
1091101	1820									11.3	10.3	34.3		

DATE	TIME	SP-GR	PH	PROT	GLUC	KETON	BILI	UROB	OB	NITR	LEU	RBC	WBC	EPCEL
1091015	0909	>1.040	5.0	2+	Negative	Trace	Negative	0.2	Negative	Negative	Trace	0-2	10-20	0-1
1091101	2103	1.021	5.0	1+	250.0	Negative	Negative	0.2	1+	Negative	Negative	2-5	0-2	6-10

DATE	TIME	STOOL-OB	FAT	RBC	WBC	PARA
1090913	2137	3+				
1091031	1654	Negative				
1091101	1552	3+				
1091105	1600			-	-	Not Found
1091220	1638	4+				

DATE	TIME	PH	PCO2	PO2	SO2	BEB	HCO3	TCO2	HGB	FIO2
1091004	1434	7.405	37.9	79.7	95.7	-1.2	23.2	24.3		
1091005	0653	7.446	34.1	67.2	93.9	-0.4	22.9	24.0		
1091005	1027	7.438	34.9	72.9	95.0	-0.4	23.1	24.2		
1091006	0802	7.477	34.2	89.9	97.6	1.7	24.7	25.7		
1091015	0227	7.488	34.4	132.2	99.2	2.5	25.5	26.5		
1091017	0549	7.542	28.5	116.0	99.1	2.6	23.9	24.8		
1091021	0527	7.439	34.7	74.4	95.3	-0.6	23.0	24.0		

DATE	TIME	PH	BEB	SCVO2	PO2	HCO3	HGB	PCO2	TCO2
1090913	1059	7.333	-2.3	93.6	75.1	23.9		46.0	25.3
1091008	0911	7.391	2.1	69.1	36.2	27.7		46.6	29.1
1091012	0803	7.420	8.4	34.5	20.3	34.8		55.0	36.5

特殊檢查

病理報告

- 活體組織切片病理報告 手術日期：**109/09/30** 病理號：**S0930897**

診斷醫師:文芙卿 報告日期:109/10/08
Pathologic diagnosis:
1. Ascending to transverse colon, laparoscopic right hemicolectomy --- Adenocarcinoma, moderately differentiated. Tumor invades mesocolic soft tissue. The appendix, ileocecal valve, bilateral cut ends and mesocolic lymph nodes (0/25) are free of tumor.
2. Lymph node, middle colic root, dissection --- Unremarkable. Negative for metastatic carcinoma (0/7).
3. Gallbladder, LC --- Cholesterol polyp.
Ancillary study for diagnosis:
1. IHC stains for MLH1, MSH2, PMS2 and MSH6 are done on section P. All above markers are preserved.
2. IHC stain for AE1/AE3 (- on target lesion) done in section Q.
Prognostic and predictive factor:
1. Tumor size: 5x3.4 cm.
2. Depth of invasion: Mesocolic soft tissue.

- 3. Histologic grade: G2: Moderately differentiated.
4. Angiolymphatic invasion: Not identified.
5. Perineural invasion: Not identified.
6. Tumor deposits (discontinuous extramural extension): Not identified.
7. Macroscopic tumor perforation: Not identified.
8. Surgical Margins:
a) Proximal Margin: Uninvolved by invasive carcinoma, distance of tumor from margin: 2 cm.
b) Distal Margin: Uninvolved by invasive carcinoma, distance of tumor from margin: 23 cm.
c) Radial or Mesenteric Margin: Uninvolved by invasive carcinoma, distance of tumor from margin: 1.5 cm.
9. Lymph node status: Uninvolved (0/32).
10. Extranodal involvement: Not applicable.
11. Tumor regression grading S/P CCRT: No known presurgical therapy.
12. Type of polyp in which invasive carcinoma arose: Not identified.
13. Additional pathologic findings: Not identified.
14. Pathological TNM Stage: pT3N0(According to the eighth edition, American Joint Committee on Cancer Staging Guidelines for Tumors).
15. TNM descriptors: Not applicable.
Gross description:
The specimen consists of 1) a segment of colon, 26 cm in length, and 5 cm in distal cut end circumference, and a segment of terminal ileum, 2.5 cm in length, and 2 cm in cut end circumference. An unremarkable appendix, 4x0.8x0.5 cm, is noted. A gray tan firm annular elevated and centrally ulcerative tumor, 5x3.4 cm, is noted, 2 cm proximal to the distal cut end. On cross section, the tumor invaded to the mesocolon with 15 mm to the radial margin grossly. The appendix, ileocecal valve, and both cut ends are free of tumor. 2) a piece of tan gray soft tissue, 2.5x2x1.1 cm, labeled as "middle colic root". 3) gallbladder, 5.5x3 cm, with wall 0.2 cm in width. Some small polyps up to 0.3x0.2x0.2 cm are noted. All for section: A) specimen 2; Representative part for section: B) specimen 3 C) proximal cut end D) distal cut end E) appendix F) ileocecal valve G) radial cut end H-P) tumor Q-S) LN.
Note:
1. The interpretation of MMR panel and recommended studies are as below:
(1) No loss of nuclear expression of MMR proteins: low probability of microsatellite instability-high (MSI-H).
(2) Loss of nuclear expression of MLH1 and PMS2: testing for methylation of the MLH1 promoter and/or mutation of BRAF is indicated (the presence of a BRAF V600E mutation and/or MLH1 methylation suggests that the tumor is sporadic and germline evaluation is probably not indicated; absence of both MLH1 methylation and of BRAF V600E mutation suggests the possibility of Lynch syndrome, and sequencing and/or large deletion/duplication testing of germline MLH1 may be indicated).
(3) Loss of nuclear expression of MSH2 and MSH6: high probability of Lynch syndrome (sequencing and/or large deletion/duplication testing of germline MSH2 may be indicated, and, if negative, sequencing and/or large deletion/duplication testing of germline MSH6 may be indicated).
(4) Loss of nuclear expression of MSH6 only: high probability of Lynch syndrome (sequencing and/or large deletion/duplication testing of germline MSH6 may be indicated).
(5) Loss of nuclear expression of PMS2 only: high probability of Lynch syndrome (sequencing and/or large deletion/duplication testing of germline PMS2 may be indicated).
(6) Please note: There are exceptions to the above IHC interpretations. These results should not be considered in isolation, and clinical correlation with genetic counseling is recommended to assess the need for germline testing.
2. This case has been peer reviewed by two doctors.
#T-59440_2 #M-82633_2 2 1105 S0000F
#T-C4560_2 #M-09183_2 0 1105 000000
#T-59200_2 #M-09183_2 0 1105 000000

活體組織切片病理報告　手術日期：**109/09/15**　病理號：**S0928662**

診斷醫師:陳韻安 報告日期:109/09/18
Pathologic diagnosis:
Colon, 60 cm from anal verge, endoscopic biopsy --- Adenocarcinoma, moderately differentiated.
Gross description:
The specimen consists of multiple pieces of tan soft tissue, up to 0.2x0.1x0.1 cm. All for section.
Microscopic description:
Section shows colon mucosa with invasive carcinoma with glandular differentiation.
Note:
1. This case has been peer reviewed by two doctors.
2. The diagnosis is concurred in intradepartment consensus meeting on 2020/09/18.
#T-59300_2 #M-81403_2 2 1340 00CM0F

其它

- **檢驗項目 :Bacterial Culture(Ordinary) 檢體:Pus(RUQ wound) 報告日期 :1091218**
附註　:
陰性結果：
陽性結果：　ISOLATED ORGANISM:　　COLONY COUNT:(　)/ml
(1) Klebsiella pneumoniae　M
(2) Escherichia coli　M
(3) Staphylococcus epidermidis　M
菌落量定義：P:Pure　H:Heavy　M:Moderate　S:Slight moderate　F:Few　E:Enrich
SUSCEPTIBILITY TEST:　S:Susceptible　I:Intermediate　R:Resistant D: Susceptible Dose Dependent (SDD) NS: Nonsusceptible

	(1)	(2)	(3)	(4)
Ampicillin				
Cefazolin	R>=64	R>=64		
Clindamycin				
Clarithromycin				
Gentamicin	I=8	S=4		
Amikacin	S=2	S=16		
Chloramphenicol				
Ampicillin/Sulbactam	R>=32	S=8		
Penicillin				
Gentamicin(120ug)				
Trimethoprim+Sulfamethoxazole	R>=320	S=20		
Vancomycin				
Oxacillin				
Cefpirome				

Ciprofloxacin	R=1	S=0.25
Minocycline		
Carbapen (IPM)	S=0.25	S=1
Cefoperazone/Sulbactam	S=16	S=8
Cefotaxime		
Ceftazidime	R>=64	S=0.5
Cefepime	S=0.25	S=0.12
Ceftriaxone	R=32	S=1
Flomoxef	I=16	I=16
Levofloxacin		
Teicoplanin		
Piperacillin/Tazobactam	R>=128	S=4
Cefmetazole		
Aztreonam		
Tigecycline		
Amoxicillin/Clavulanic acid		
Cefoxitin	R=32	R>=64
Metronidazole		
Carbapen (MPM)		
Erythromycin		
Moxifloxacin		
Linezolid		
Ertapenem	S=0.25	S=0.12
Colistin		
Daptomycin		

REMARK

注意事項： For Enterobacteriaceae :

1.對於腸內菌屬的敏感性試驗，cefepime若採用CLSI M100-24的判讀標準，當試驗結果為敏感性時，其建議治療劑量為1gm Q12H；若以最低抑制濃度（MIC）的方法，結果為敏感劑量依賴性（susceptible-dose dependent；SDD）時，當MIC為 4 ug/mL時則建議劑量為1gm/Q8H或2gm/Q12H，若是MIC為8 ug/mL時或採用紙錠擴散法結果為SDD，則建議治療劑量為2gm/Q8H。

2.Cefazolin predict results for the oral cephalosporins agents (cefaclor, cephalexin) when used for therapy of uncomplicated UTIs due to E. coli, K. pneumoniae, and P. mirabilis.

3.Cefoperazone/Sulbactam目前並無國際性的判讀標準，現行引用文獻的判讀規範，僅供臨床參考。

For Staphylococcus spp. :

1.對penicillin呈感受性的葡萄球菌亦會對FDA認可用於治療葡萄球菌感染的其他青黴素類、beta-lactam/beta-lactamase 抑制劑的合併藥物、cephems及carbapenems呈感受性。

2.對pencillin呈抗藥性，但對oxacillin呈感受性的菌株將對可被penicillinase破壞的青黴素類藥物也呈抵抗性；但對不被beta-lactamase破壞的青黴素類，beta-lactam/beta-lactamase抑制劑合併藥物、cephems及carbapenems呈感受性。

3.對於葡萄球菌屬不建議單獨使用rifampin治療。

4.Aminoglycosides 僅可與其他有效的藥物合併使用。

出院時情況

- 3.改門診治療

出院指示

藥名	劑量	劑量單位	用法	頻次	數量	數量單位	現況	天數	單位
Loperamide cap 2mg	1	CAP	PO	TID	21	CAP	TH	7	天
Nebilet tab 5mg	0.5	TAB	PO	QD	4	TAB	TH	7	天
(口)Takepron OD tab 30mg	1	TAB	PO	QDAC	7	TAB	TH	7	天
200mg Entresto tab	1	TAB	PO	BIDPC	14	TAB	TH	7	天
Tamso SR cap 0.2mg	2	CAP	PO	QD	14	CAP	TH	7	天
25mg Utapine tab	1	TAB	PO	QN	7	TAB	TH	7	天
Forxiga tab 10mg	1	TAB	PO	QD	7	TAB	TH	7	天
Acetylcysteine tab	1	TAB	PO	QN	7	TAB	TH	7	天
Zulitor tab 4mg	1	TAB	PO	QD	7	TAB	TH	7	天

出院計劃

主治醫師 2222H 林俊[illegible] / 住院醫師 9761G 王文謙

正式摘要(電子簽章：000000206187179)

W55 / W55

doctorRank:B
